# Rapid and robust quantitative cartilage assessment for the clinical setting: deep learning-enhanced accelerated T2 mapping

**DOI:** 10.1007/s00256-025-05034-w

**Published:** 2025-09-18

**Authors:** Laura Carretero-Gómez, Florian Wiesinger, Maggie Fung, Bruno Nunes, Valentina Pedoia, Sharmila Majumdar, Arjun D. Desai, Anthony Gatti, Akshay Chaudhari, Eugenia Sánchez-Lacalle, Norberto Malpica, Mario Padrón

**Affiliations:** 1 GE HealthCare, Madrid, Spain; 2 Medical Image Analysis and Biometry Lab, Rey Juan Carlos University, Madrid, Spain; 3 GE HealthCare, Munich, Germany; 4 GE HealthCare, New York, NY USA; 5 GE HealthCare, San Ramon, CA USA; 6 Department of Radiology and Biomedical Imaging, San Francisco (UCSF), University of California, San Francisco, CA USA; 7 Radiology, Stanford University, Stanford, CA USA; 8 Department of Radiology, Clínica CEMTRO, Madrid, Spain

**Keywords:** Quantitative MRI, T2 mapping, Deep learning, Knee cartilage, Repeatability, Reproducibility

## Abstract

**Objective:**

Clinical adoption of T2 mapping is limited by poor reproducibility, lengthy examination times, and cumbersome image analysis. This study aimed to develop an accelerated deep learning (DL)-enhanced cartilage T2 mapping sequence (DL CartiGram), demonstrate its repeatability and reproducibility, and evaluate its accuracy compared to conventional T2 mapping using a semi-automatic pipeline.

**Methods:**

DL CartiGram was implemented using a modified 2D Multi-Echo Spin-Echo sequence at 3 T, incorporating parallel imaging and DL-based image reconstruction. Phantom tests were performed at two sites to obtain test–retest T2 maps, using single-echo spin-echo (SE) measurements as reference values. At one site, DL CartiGram and conventional T2 mapping were performed on 43 patients. T2 values were extracted from 52 patellar and femoral compartments using DL knee segmentation and the DOSMA framework. Repeatability and reproducibility were assessed using coefficients of variation (CV), Bland–Altman analysis, and concordance correlation coefficients (CCC). T2 differences were evaluated with Wilcoxon signed-rank tests, paired *t* tests, and accuracy CV.

**Results:**

Phantom tests showed intra-site repeatability with CVs ≤ 2.52% and T2 precision ≤ 1 ms. Inter-site reproducibility showed a CV of 2.74% and a CCC of 99% (CI 92–100%). Bland–Altman analysis showed a bias of 1.56 ms between sites (*p* = 0.03), likely due to temperature effects. In vivo, DL CartiGram reduced scan time by 40%, yielding accurate cartilage T2 measurements (CV = 0.97%) with no significant differences compared to conventional T2 mapping (*p* = 0.1).

**Conclusions:**

DL CartiGram significantly accelerates T2 mapping, while still assuring excellent repeatability and reproducibility. Combined with the semi-automatic post-processing pipeline, it emerges as a promising tool for quantitative T2 cartilage biomarker assessment in clinical settings.

## Introduction

Osteoarthritis (OA) is the most prevalent type of arthritis and a significant health concern for the aging population, affecting approximately 73% of adults over 55 years of age [1]. OA is a degenerative disease characterized by morphological and biochemical alterations involving all anatomical components of the joint. A hallmark of knee OA is the breakdown of articular cartilage. Early biochemical changes in the proteoglycan and collagen matrix precede the development of focal defects, potentially leading to more diffuse cartilage loss associated with established OA [2].

Magnetic resonance imaging (MRI)-based compositional analysis enables the detection of these biochemical and microstructural changes at early stages of OA, before they become visible with conventional structural MRI sequences and arthroscopy [3]. Among the various available methods [2], T2 mapping is currently the best-established and the only commercially available MRI technique for assessing cartilage composition, with a large body of supporting literature mostly focusing on the knee articular cartilage [4, 5]. T2 values reflect the water content, collagen integrity, and fiber orientation within the extracellular matrix, with longer T2 values being indicative of cartilage degeneration [6, 7]. Besides diagnosis, T2 mapping can also be used to monitor the response to cartilage repair treatments [8, 9]. However, the lack of standardization to reduce measurement variability and achieve comparable outcomes across different scanners is hindering its clinical translation [3].

To address this challenge, the Musculoskeletal Biomarkers Committee of the Quantitative Imaging Biomarkers Alliance (QIBA) developed a dedicated QIBA profile for cartilage compositional imaging [3]. This profile provides standardized guidelines and recommendations to enhance reproducibility of compositional cartilage imaging techniques. Notably, it establishes that cartilage T1ρ and T2 values can be reliably measured at 3 T MRI with a within-subject coefficient of variation of 4–5%, thus improving consistency and comparability across clinical sites.

The classical in vivo approach to T2 mapping employs a two-dimensional multi-echo spin-echo (MESE) pulse sequence that acquires multiple, equally spaced spin echoes of the same k-space line from a single RF excitation [10]. A widely used clinical implementation of this technique is the CartiGram sequence (GE HealthCare, Waukesha, WI). However, fully sampling the spatiotemporal T2 relaxation in this manner is still time-consuming, highlighting the need for further acceleration.

Parallel imaging is a well-established method to accelerate lengthy acquisitions by reducing the number of k-space readouts via extra coil sensitivity encoding [11, 12]. However, this acceleration typically comes at the cost of reduced signal-to-noise ratio (SNR), which scales inversely with the square root of the acceleration factor and the coil geometry factor [12]. Recently introduced deep learning (DL)-based image reconstruction methods have demonstrated significant improvements in image quality (i.e., SNR and sharpness) as well as image encoding efficiency (i.e., scan time and coverage) [13]. Such novel DL reconstruction method can potentially overcome noise-enhancement intrinsic to parallel imaging, thereby making T2 mapping more feasible in clinical practice.

In addition to acquisition burdens, the T2 mapping workflow often involves time-consuming manual post-processing steps for extracting cartilage T2 values from relevant anatomical regions. Automation of segmentation, registration, and quantification would significantly streamline this process, enabling accurate and robust T2 cartilage biomarker assessment in clinical settings [14].

The goal of this study was threefold: (1) to implement a novel T2 mapping sequence combining parallel imaging acceleration with DL-based reconstruction, (2) to demonstrate its robustness in terms of intra-site repeatability and inter-site reproducibility using a calibrated T2 reference phantom, and (3) to integrate a semi-automated analysis pipeline for knee relaxometry and assess the performance of this DL-enhanced CartiGram sequence relative to conventional T2 mapping in patients.

## Materials and methods

### Study design

A parallel imaging-accelerated version of the CartiGram sequence (GE HealthCare, Waukesha, WI), an MESE sequence that acquires up to eight echoes per excitation, was implemented for rapid knee cartilage T2 mapping. Acceleration was achieved using ARC (autocalibrating reconstruction for cartesian imaging) [15]. To improve SNR and image sharpness, the reconstruction incorporated the commercially available AIR™ Recon DL reconstruction algorithm [13] (GE HealthCare, Waukesha, WI). This DL method operates on raw k-space data and uses a convolutional neural network (CNN) to reconstruct images with minimal noise and ringing artifacts while preserving edge detail. The CNN was trained using supervised learning on a large, independent dataset comprising high-quality reference images and their synthetically degraded counterparts spanning multiple anatomies and imaging conditions. Extensive data augmentation was applied to promote generalizability and robustness. Notably, no CartiGram data were included in the training set.

MR imaging experiments were conducted on two GE HealthCare 3 T MR systems installed at two different sites, each using a dedicated Tx/Rx knee coil: Site 1 – 3 T SIGNA™ Architect with 18-Ch Tx/Rx knee coil (QED, Hannover, Germany); and Site 2 – 3 T Discovery™ MR750w with 8-Ch HD Tx/Rx Knee coil (Invivo, Gainesville, FL).

### Phantom study

**Fig. 1:**
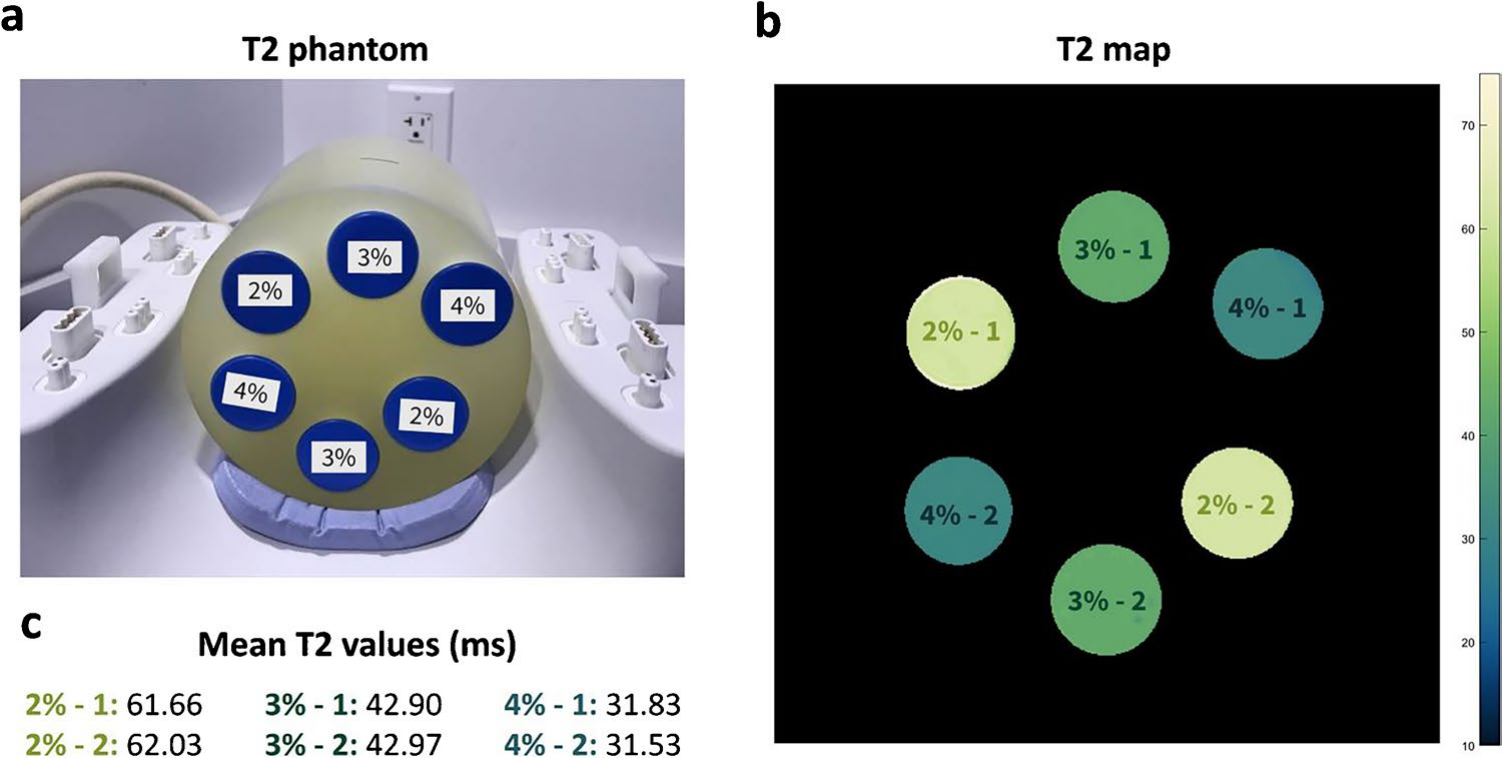
**a** Photo of T2 phantom used in this study showing agarose concentration labels. **b** Fitted T2 map from the SE experiment. **c** Mean T2 values computed at each vial. Upper vials are denoted as “ − 1” and lower as “ − 2”

A phantom consisting of three pairs of oppositely arranged tubes with different agarose concentrations (i.e., 2%, 3%, and 4%, weight/volume) fixed in a cylinder holder was used in this study (Fig. 1**a**) (custom-manufactured by Phantom Laboratory, Salem, NY), mimicking knee tissue properties. Reference T2 values were obtained from a spin-echo (SE) experiment run at Site 2, consisting of ten separate single echo acquisitions, one for each echo time (TE) [10–100 ms] using a long repetition time (TR) of 15 s to eliminate T1 relaxation effects (Fig. 1**b, c**). The phantom travelled between sites to complete the experiments explained below.

To evaluate the impact of acceleration on T2 measurements, CartiGram scans with ARC acceleration factors of 1 (i.e., no acceleration), 2, 3, and 4 were acquired and reconstructed with and without DL-enhancement at Site 1 (TE = 6.8–54.4 ms with echo spacing (esp) = 6.8 ms, TR = 1200 ms, 10 axial slices, FOV = (16 cm)^2^, res = (0.3125 mm)^2^ × 3.6 mm). This analysis enabled the selection of an optimal configuration that balanced scan time and SNR: ARC acceleration factor = 3 combined with DL-based reconstruction, which was subsequently used for the remaining phantom experiments as well as for the in vivo patient study.

To assess intra-site repeatability and inter-site reproducibility, the phantom was scanned multiple times on different days at both sites using the optimized protocol, maintaining the same positioning and orientation within the coil. To minimize temperature-induced variability, the phantom was stored in the scanner room at least overnight prior to each scan. Room temperature was recorded, and a temperature-sensitive sticker was affixed to the phantom to monitor pre- and post-scan temperature. The protocol was also tested with the phantom placed at three coil positions (left 70 mm, isocenter, and right 70 mm) to simulate knee laterality.

### Patient study

After institutional review board approval and written informed consent, 43 patients (mean age 36 ± 12 years; 11 female; mean weight 75 ± 15 kg) were prospectively recruited from institution’s daily schedule for routine clinical knee MRI. A total of 52 compartments were analyzed (21 patellofemoral and 31 femoral), as nine subjects had both knees or both compartments imaged. Thirty-five patients (81%) had previously undergone autologous chondrocyte implantation for focal cartilage defects, while the remaining presented with other knee pathologies, such as prior anterior cruciate ligament reconstruction and/or meniscal surgery, without cartilage repair. Only patellar and femoral cartilage were evaluated in this study. Most cases exhibited moderate to severe cartilage damage at the treated site (Outerbridge grade III–IV), whereas radiographic osteoarthritis was minimal (Kellgren–Lawrence grade 0–1), reflecting a population with localized cartilage pathology rather than diffuse degenerative disease. Patients were scanned at Site 1 adding to the routine knee clinical protocol a sagittal fat-suppressed 3D FSE CUBE sequence (TE/TR = 60/1500 ms, FOV = (16 cm)^3^, resolution = (0.3125 mm)^2^ × 0.6 mm, scan time = 5 min); and two 2D CartiGram sequences, one with no acceleration and conventional reconstruction (conventional CartiGram) and one using the previously optimized configuration with ARC acceleration factor = 3 and DL reconstruction (DL CartiGram), in sagittal orientation for femoral and axial for patellar compartments. To reduce conventional CartiGram scan time, only the 13 slices best covering the targeted region (condyle or patella) were acquired with both sequences (TE = 6.8–54.4 ms, esp = 6.8 ms, TR = 1200 ms, FOV = (16 cm)^2^, res = (0.3125 mm)^2^ × 3.6 mm, scan time = 5:10 for conventional vs 3:00 min for DL CartiGram (with no-phase-wrap factor = 1.6)).

### Image processing

#### Phantom

A semi-automatic segmentation method was used to define the regions of interest (ROIs) for each vial. This was achieved by removing the background using a user-adjustable threshold for masking, followed by identification of the six connected components.

T2 maps were then obtained by a voxel-wise mono-exponential fitting of the segmented vials. The first echo of the 2D MESE T2 images was skipped from the fitting to minimize the bias introduced by stimulated echoes [16]. Following the fitting, T2 maps were eroded at the periphery to reduce partial volume and ringing effects. For each ROI/vial, the mean and standard deviation (SD) of T2 values were calculated.

In accordance with a recently published recommendation for harmonizing the color schemes used in MR relaxometry, the logarithm-processed Navia color-map was applied to visualize the T2 maps [17].

**Fig. 2:**
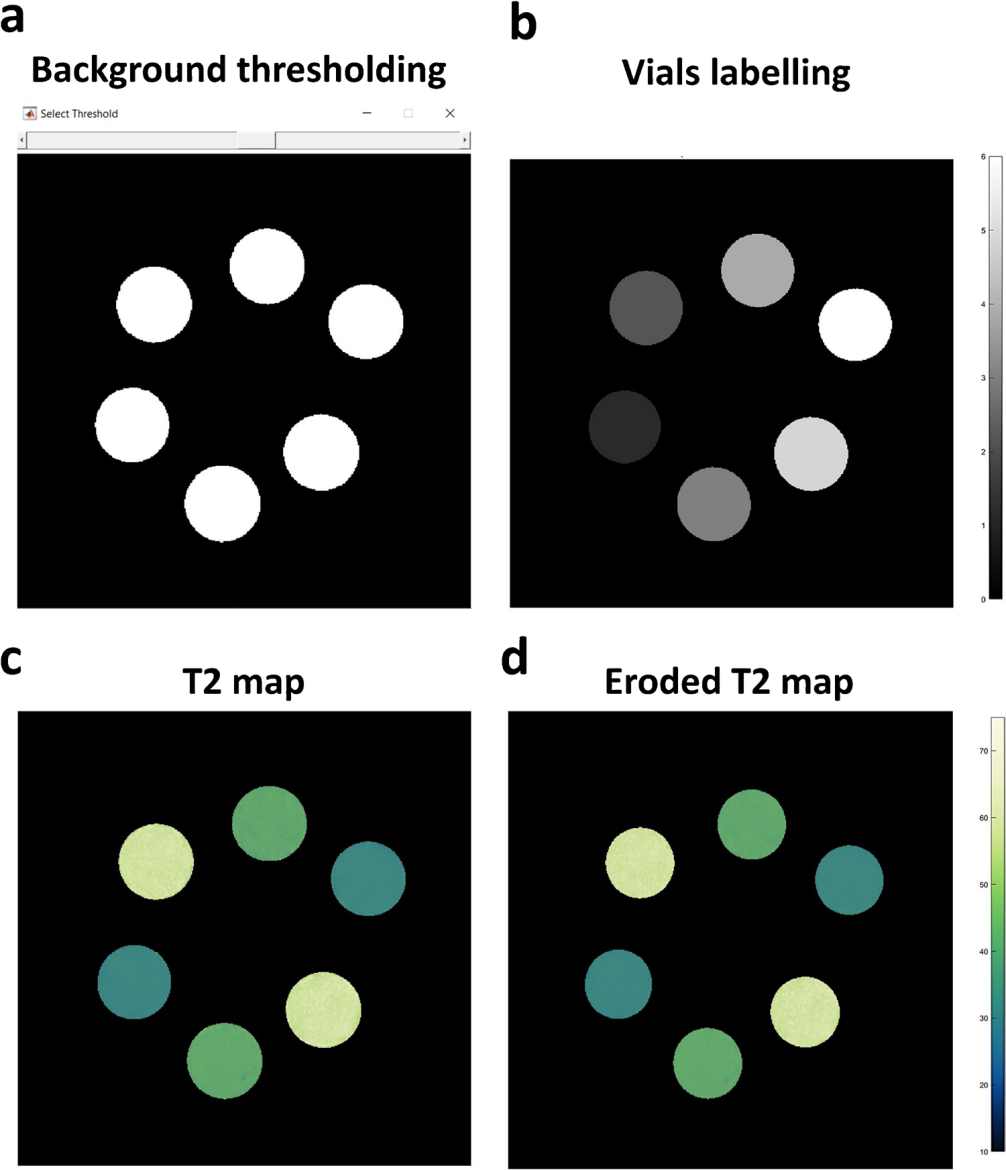
Phantom image processing in MATLAB. **a** Background was masked with a user-adjustable threshold. **b** Vials were automatically extracted and labelled. **c** T2 values were computed with mono-exponential fitting within the segmented vials. **d** Periphery of each vial was eroded to avoid edge effects before computing T2 mean and standard deviation values.

All processing steps were performed using MATLAB R2022a (MathWorks, Natick, MA) (Fig. 2).

#### Patients

**Fig. 3:**
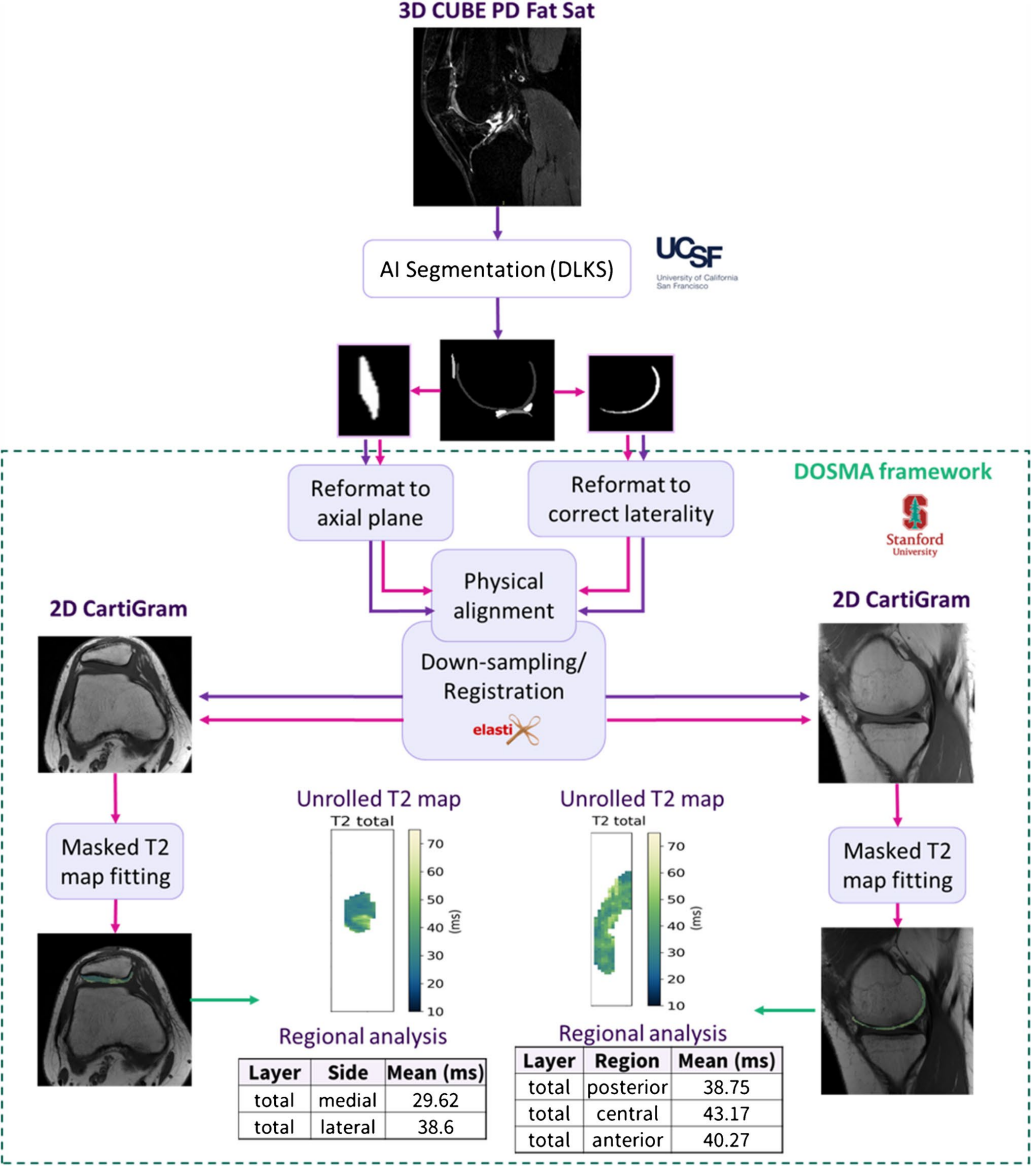
Pipeline for patient image processing based on DOSMA framework. Cartilage segmentation was performed on CUBE scans using an external AI-based model (DLKS). CUBE, conv., and DL CartiGram were sequentially scanned keeping the same field of view; this likely explains that no shearing was needed during registration, and a rigid transformation was sufficient to align the images

For human subjects, a Python-based pipeline was developed using the Deep Open-Source Medical Analysis (DOSMA) framework [18] (Fig. 3).

The DL Knee Segmentation (DLKS) model [19, 20] was employed to automatically segment the cartilage from the 3D CUBE images into binary masks with a different label for each extracted cartilaginous compartment. These masks were then provided to the pipeline as additional input. Since the analysis focused on femoral cartilage (FC) and patellar cartilage (PC), the corresponding labels were automatically extracted from the masks.

Since only one region of interest (either a single condyle or the patella) was analyzed per case to meet scan time limitations in routine clinical practice, spatial coverage differed between the CUBE sequence (which covers the entire knee) and the CartiGram acquisitions (which target only the selected region). As a result, the volumes had different shapes and sizes. Additionally, in femoral cases, CartiGram slices may begin at the level of the anterior cruciate ligament (ACL)—for example, when imaging the lateral femoral condyle (LFC) in the left knee or the medial femoral condyle (MFC) in the right knee—while CUBE would start from the opposite condyle and span the full extent of the knee, based on a right-to-left prescription from the patient’s perspective.

To address these coverage discrepancies and enhance pipeline robustness, an additional step was included to automatically reformat CartiGram and adjust laterality, ensuring the acquisition does not start at the ACL. The CUBE volume and its corresponding femoral mask were reformatted to match the same laterality. For patellar cases, the CUBE volume and patellar mask were reformatted to the axial view. Once all inputs had consistent laterality, an initial physical alignment [21, 22] was implemented to match the spatial coordinates of the CUBE volume and mask with those of the CartiGram. This served to initialize the registration and improve its performance.

The CUBE volume was then 3D rotated, translated, and isotropically scaled to align with the first echo of the CartiGram sequence. Given its higher and more isotropic resolution, CUBE was designated as the “moving” image, and CartiGram as the “fixed” image. Mattes mutual information metric was used for this multimodal registration. The resulting transformation was applied to the mask, so that both CUBE and FC/PC were downsampled to CartiGram space. Registration parameters were refined and validated by fusing the CUBE and mask over the CartiGram datasets and visually checking the accuracy of the cartilage alignment. Registration across echoes in CartiGram datasets was unnecessary, as the echo images are intrinsically aligned in MESE acquisitions. All the registration/transformation operations were performed using Elastix [23] and Transformix [23] libraries supported by DOSMA.

For the remaining steps, cartilage analysis and visualization functions available in DOSMA were used and adapted as follows. To speed up computation, mono-exponential T2 fitting was performed within each masked compartment, excluding the first echo to account for stimulated echoes. The resulting T2 maps were projected onto an unrolled [24] 2D axial view for FC and coronal view for PC, using the logarithm-processed Navia color-map. Parametric analysis with subregion division was performed automatically; FC was divided into three layers (total, deep, and superficial) and six subregions (anterior/central/posterior for the medial/lateral sides). PC was divided into the same three layers and two subregions (medial/lateral sides). Cropped T2 maps were automatically saved in DICOM format, for convenient viewing and fusion with the CartiGram volumes.

The proposed pipeline was applied to both CartiGram acquisition, conventional and DL CartiGram, to estimate T2 maps and compare the mean T2 values across subregions in the total cartilage layer (bulk). For FC, as just one condyle was imaged per case, medial and lateral values were averaged accounting for the number of voxels measured, yielding three final subregions: anterior, central, and posterior.

### Statistical analysis

#### Phantom

Coefficients of variation (CVs) were calculated to evaluate intra-site repeatability in phantom scans. Measurements from different coil locations were considered separately when calculating the CVs. T2 precision was estimated using the median of scan–rescan standard deviations. For inter-site reproducibility, CVs and Bland–Altman (BA) [25] analysis were performed. Concordance correlation coefficients (CCC) [26] and their corresponding confidence intervals (CCC-CI) were also calculated to assess reproducibility and agreement between sites, as well as between each site and the reference SE values. Differences in T2 values were assessed with Wilcoxon signed-rank tests.

#### Patients

Accuracy CV for DL CartiGram in cartilage was calculated by comparing mean total T2 values with those obtained from conventional CartiGram. A paired *t* test was conducted to compare both techniques. Bias and Limits of agreement values were calculated with the methods of BA with 95% limits of agreement (LOA).

All statistical tests were performed at a significance level of 0.05.

## Results

### Phantom

**Fig. 4:**
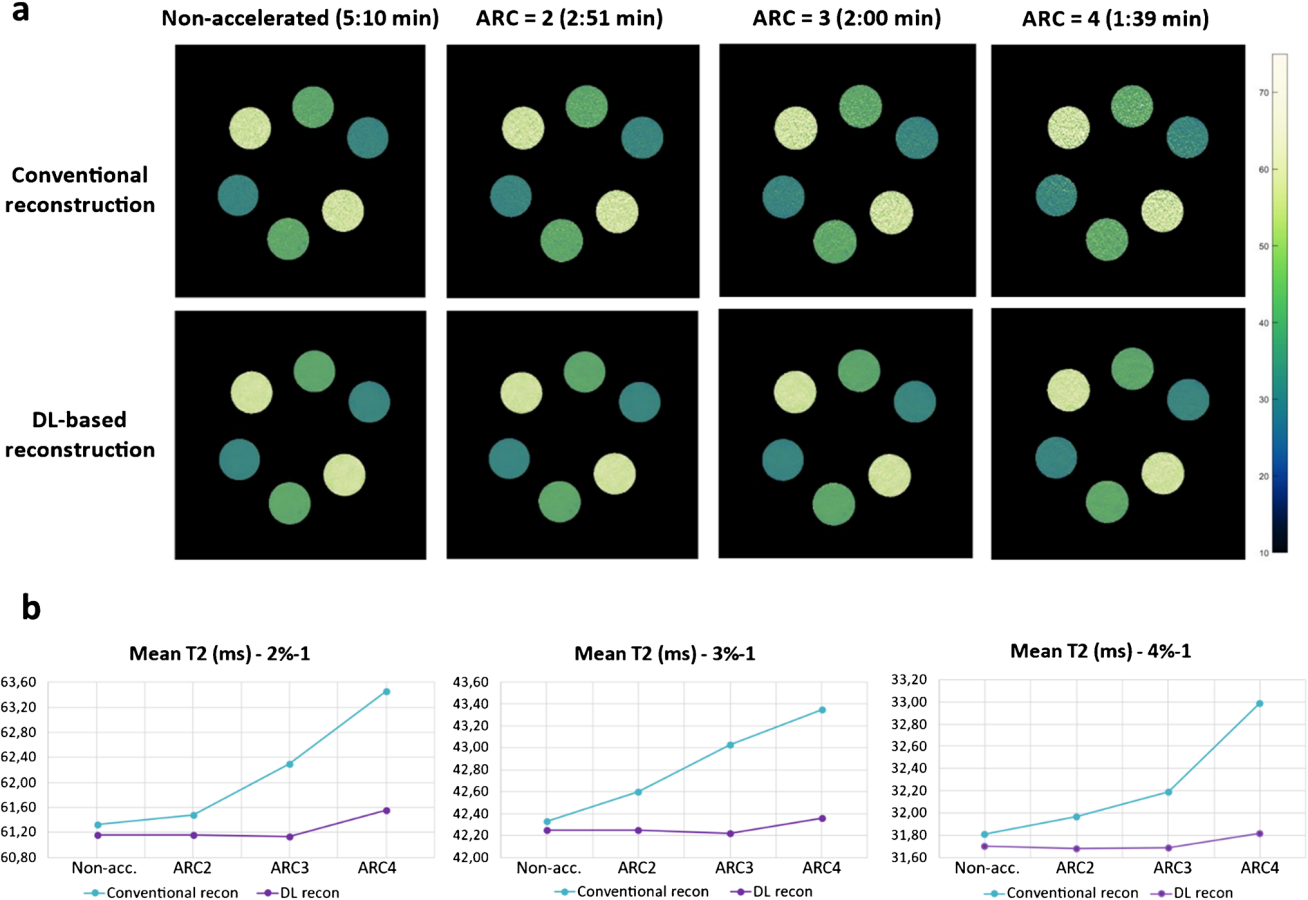
**a** T2 maps from phantom testing with different ARC acceleration factors (ARC), showing the scan time reduction at higher factors and the denoising with DL reconstruction. **b** Scatter plots comparing mean T2 values obtained at different acceleration factors with conventional (blue) versus DL (purple) reconstruction methods for each upper ROI (2%−1, 3%−1, 4%−1), including non-accelerated (non-acc.) sequence as reference values

By using higher ARC acceleration factors, a substantial scan time reduction was achieved, up to 70% for the highest acceleration factor of 4. Reduced SNR, originating from fewer k-space samples and g-factor noise amplification, was effectively recovered using DL reconstruction, as can be seen from the resulting T2 maps (Fig. 4a), which otherwise would cause overestimation of T2 values due to the elevated noise floor [27]. Comparing mean T2 values measured at the different vials, DL reconstruction provides more consistent values across different acceleration factors compared to conventional reconstruction thanks to denoising (Fig. 4b).

By visually inspecting the plots and the denoised T2maps, an ARC acceleration factor of 3 was found to provide the best trade-off between SNR and scan time reduction. This supported its selection as the optimized configuration for the repeatability/reproducibility phantom experiments and in vivo scanning. Although ARC acceleration factor of 4 also allowed for robust T2 quantification, it introduced more pronounced residual aliasing artifacts and noise amplification [28].

For the scan-rescan phantom experiments, the scanner room temperatures were approximately 21.8 °C for Site 1 and 21.3 °C for Site 2. Phantom temperature sticker showed temperatures increasing, from the beginning to the end of scanning, ranging from 18 to 22 °C at Site 1 and 20–24 °C at Site 2.

The average intra-site CV was 1.6% at Site 1, with a T2 precision of 0.62 ms, and 2.52% at Site 2, with a T2 precision of 1 ms, suggesting minimal measurement variability within each site (Table 1 a).

**Table 1:** Intra- (bottom row) and inter-site CVs (%) for phantom scans at each vial. Showing computed mean T2 values ± standard deviation (SD) (ms) and T2 precision (ms) for each site

a) Mean T2 ± SD (ms) (above) and intra-site CV (%) (below) calculated for phantom measurements at each vial								
	2% – 1	2% – 2	3% – 1	3% – 2	4% – 1	4% – 2	Average	T2 precision (ms)
Site 1	62.07 ± 0.6	62.21 ± 0.94	42.83 ± 0.44	42.66 ± 0.94	31.87 ± 0.64	31.49 ± 0.59		
	0.96	1.51	1.03	2.21	1.99	1.88	**1.6**	**0.62**
Site 2	59.4 ± 1.63	59.77 ± 1.37	41.15 ± 1.09	41.92 ± 0.9	30.79 ± 0.91	30.71 ± 0.72		
	2.74	2.3	2.64	2.14	2.94	2.33	**2.52**	**1**
b) Inter-site CV (%) for T2 phantom measurements at each vial								
	2.99	2.78	2.81	2.35	3.04	2.46	**2.74**	

**Fig. 5:**
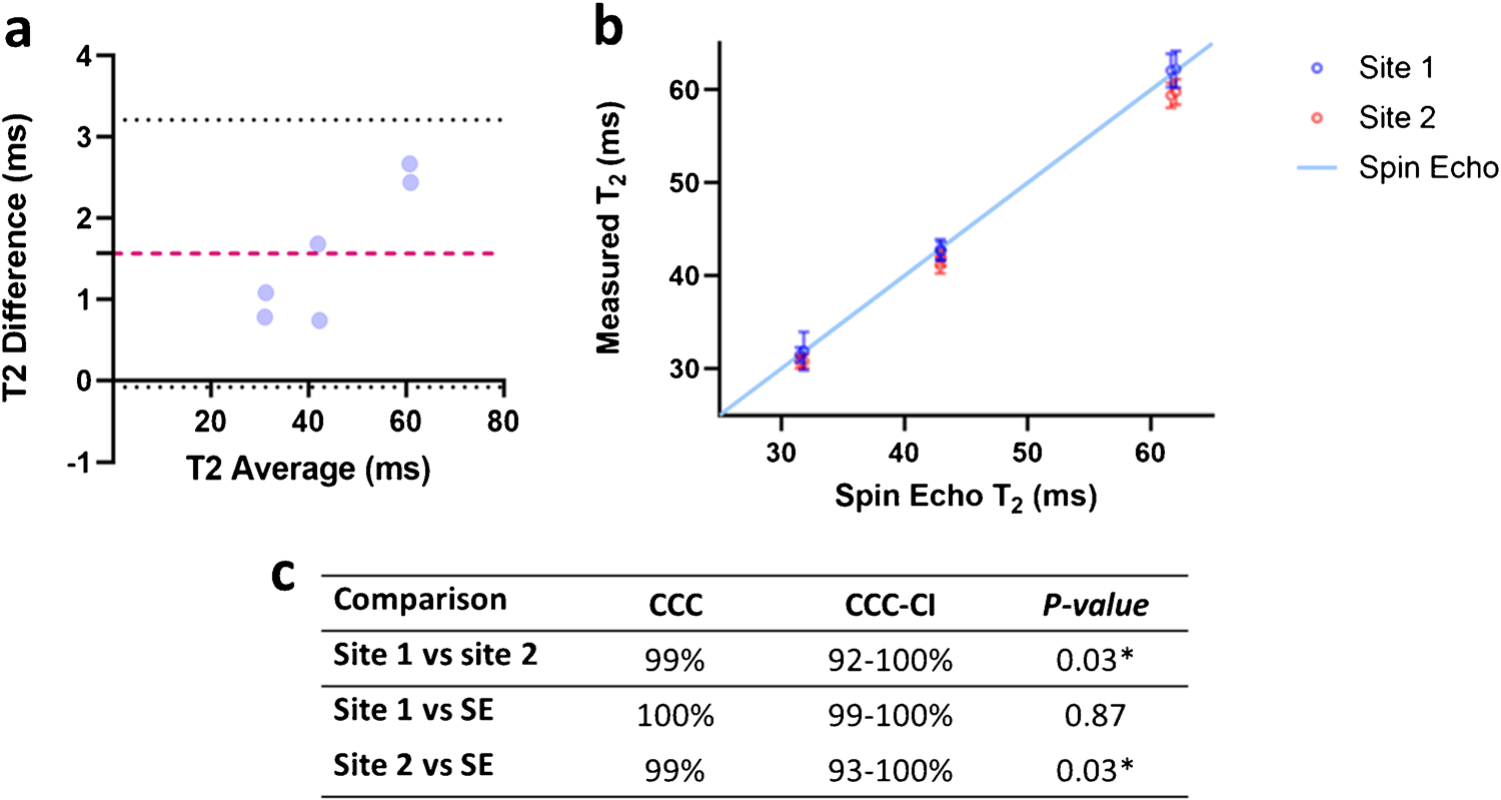
**a** Bland–Altman plot showing the reproducibility of T2 measurements between sites. Differences were computed by subtracting Site 2 measurements from their corresponding Site 1 measurements. The dashed pink line represents the mean of the differences (bias), and the dotted black lines indicate the upper and lower LOA. There was a systematic error observed. **b** Mean T2 and its standard deviation for both sites in comparison to the values obtained with the SE sequence (identity line). **c** Statistical results and concordance correlation coefficient (CCC), with its corresponding 95% confidence interval (CCC-CI), from the intra-site and site to SE reference comparisons. * indicates statistically significant

Bland–Altman analysis revealed a systematic bias in T2 measurements between sites, particularly at higher T2 vials, with Site 1 yielding higher values than Site 2. The mean inter-site bias was 1.56 ms, and the difference was statistically significant (*p* = 0.03) (Fig. 5a, c).

Furthermore, when comparing estimated values at each site with the reference values from the spin-echo (SE) experiment, a significant difference was observed for Site 2 (*p* = 0.03), which underestimated T2 values. In contrast, Site 1 demonstrated higher accuracy, providing estimates closely aligned with the SE reference (Fig. 5b, c).

Despite these differences, overall inter-site reproducibility remained high, with an average CV of 2.74% and a concordance correlation coefficient (CCC) of 99% (92–100% CI), indicating strong agreement between sites (Table 1 b, Fig. 5c).

### Patients

In the in vivo experiment, a substantial scan time reduction of approximately 40% was achieved using the selected ARC factor of 3. However, the extent of scan time reduction was constrained by the required use of a no-phase-wrap factor to avoid popliteal pulsation and aliasing artifacts, a limitation that was not present during the acceleration assessment in the phantom study.

**Fig. 6:**
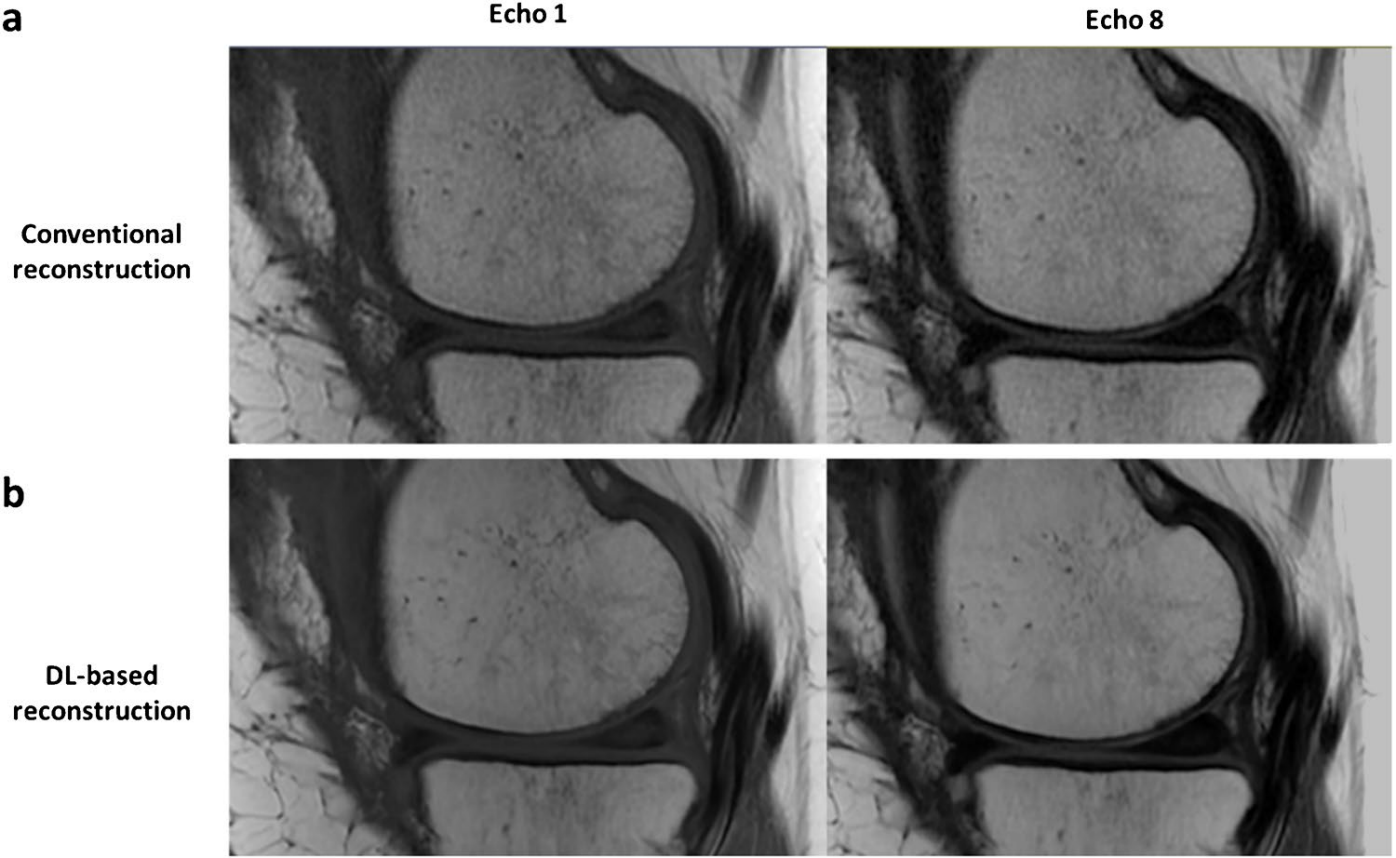
Image quality comparison of accelerated CartiGram with ARC acceleration factor = 3 using **a** conventional and **b** DL-based reconstruction, for the first and last echoes

DL-based reconstruction improved SNR in the accelerated CartiGram acquisition compared to conventional reconstruction, as visually observed in Fig. 6. Likewise, DL reconstruction provided more robust T2 maps, with reduced standard deviation, similar to the non-accelerated ones, with an average accuracy CV = 0.97%.

**Fig. 7:**
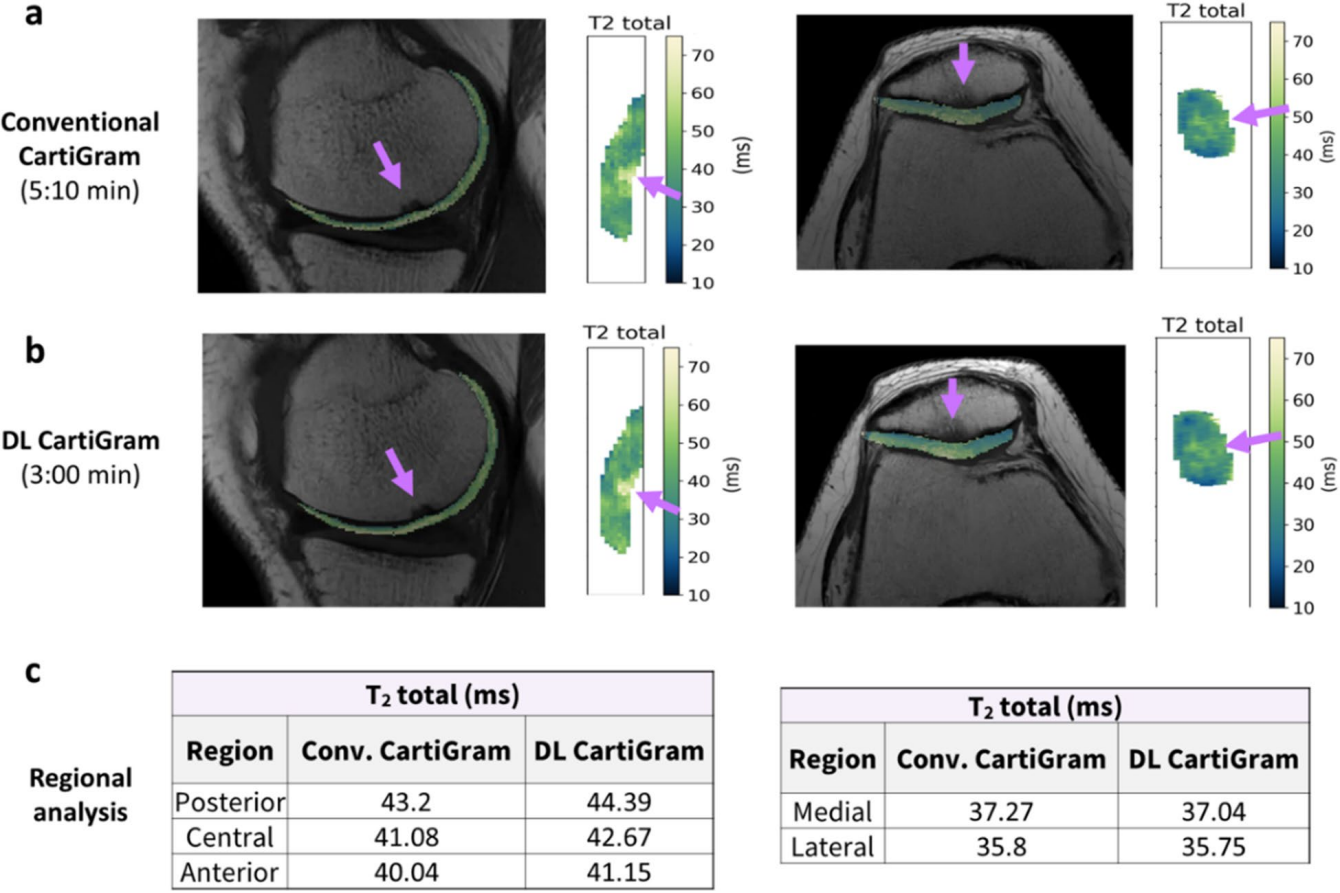
Visual comparison of femoral and patellar cropped T2 maps and unrolled maps for **a** conventional CartiGram vs** b** DL CartiGram. The femoral compartment is projected into axial plane, while patellar cartilage is unrolled into coronal view. The femoral case shows a cartilage lesion with higher T2 values, while the patellar corresponds with a 1-year follow-up study after cartilage implantation. **c** Comparison of bulk T2 values estimated through automatic regional analysis

It took on average 2 min for a trained operator to run the complete relaxometry pipeline for a single case. A total of 135 measurements from the lateral and medial sides of 21 patellar cases, and from the anterior, central, and posterior regions of 31 femoral compartments were included in this study. Thanks to the projected view combined with the cropped T2 map fused onto the CartiGram sequence generated by the pipeline, early assessment of cartilage lesions and a better understanding of cartilage repair tissue can now be achieved (Fig. 7).

**Fig. 8:**
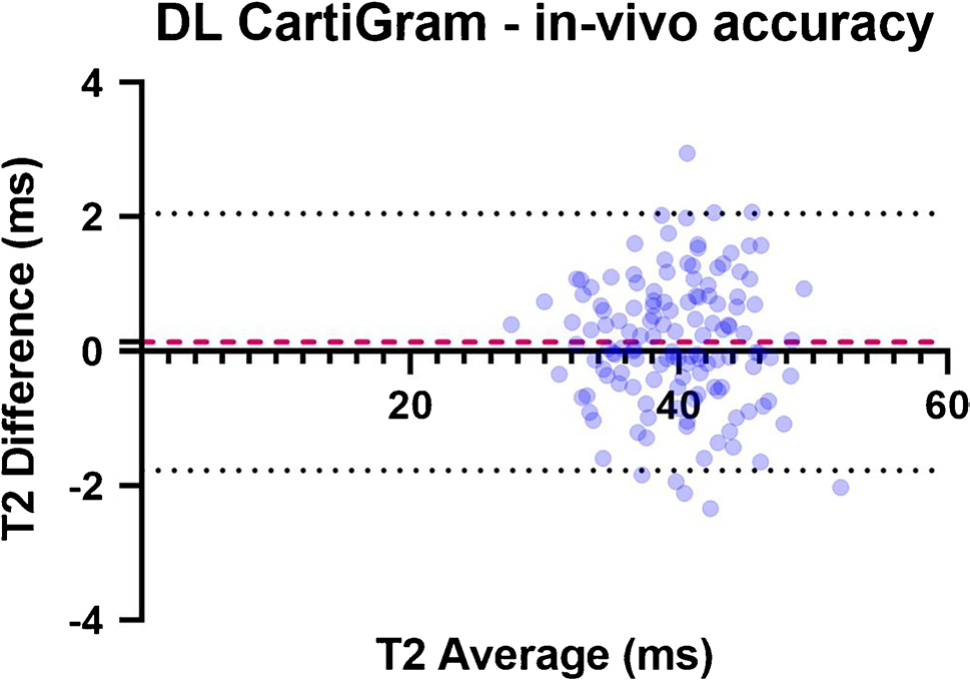
Bland–Altman plot showing the in vivo accuracy of DL CartiGram. BA differences were computed by subtracting DL CartiGram total T2 measurements from their corresponding measurements from conventional CartiGram. The dashed pink line represents the mean of the differences (bias), and the dotted black lines indicate the upper and lower limits of agreement (LOA). It is expected that the LOA Lines include 95% of differences between the two acquisition methods

Paired *t* test showed no statistically significant difference between the T2 values obtained using the two CartiGram acquisitions (*p* = 0.1). Bland–Altman analysis also revealed no significant bias (i.e., ≤ 0.15 ms) between the conventional and DL-enhanced CartiGram acquisitions. The distribution of the values was randomly oriented around the zero-difference line, with LOA lines containing 96% of the values (Fig. 8).

## Discussion

To effectively translate T2 mapping into the clinical setting, it is crucial to establish its accuracy and robustness, together with a user-friendly and intuitive workflow. In this study, we implemented a novel 2D MESE technique for knee cartilage T2 mapping through ARC acceleration combined with DL reconstruction enhancement. We validated this DL CartiGram sequence by assessing the repeatability and reproducibility of T2 measurements in a multi-site phantom study and comparing in vivo cartilage T2 measurements with conventional CartiGram.

DL CartiGram demonstrated excellent T2 precision (0.62–1 ms) in the intra-site phantom repeatability experiments, with an average CV of 1.6–2.52%, which aligns with previous studies reporting CVs ≤ 2.45% and CVs ≤ 2.8% for single site studies [4, 29]. Our findings are also in agreement with the accepted variability range according to the QIBA profile [3] which states that test–retest variability (nonlongitudinal) of cartilage T2 and T1 *ρ* values are measurable at the knee with a within-subject coefficient of variation of 4–5%. Our inter-site reproducibility was also excellent, with an average CV of 2.74%, outperforming previous reports of multi-site studies, which showed an average inter-site reproducibility of 5.96% with different vendors [29] and 4.1% for the same vendor [4]. Our excellent repeatability and reproducibility can be attributed to the reduced noise floor provided by the DL reconstruction method, which minimizes bias and thereby enhances T2 precision compared to T2 mapping techniques with conventional reconstruction.

While there was a high inter-site concordance (CCC = 99%, (92–100%)), Site 1 T2 values matched the SE reference values closely whereas Site 2 showed slightly shorter T2 values (*p* = 0.03). There are several factors that may affect relaxation time quantification such as system hardware, RF coil configuration, and environmental fluctuations. The two systems used in this study had same specifications in terms of peak gradient amplitude, gradient slew rate, RF transmit, and bore size, which resulted in the same pulse sequence timings (i.e., RF pulse width and TE spacing). Although systems were very similar, the performance of the different Tx/Rx RF coils used is expected to impact T2 measurements as they vary with respect to the number and shape of RF coil elements (sensitivity profiles).

Instead, the main difference between sites that can explain the T2 differences observed was the phantom temperature, as Site 2, with shorter relaxation times, recorded higher temperatures up to 24 °C. To investigate the impact of temperature on T2 measurements, we conducted a controlled experiment by placing the phantom outside the scanner room at Site 1 until it warmed up to 24 °C and then measured T2 values. The phantom was then left in the scanner room for 2 days until it reached 20 °C, and T2 values were measured again. We calculated the temperature coefficient for each vial, which ranged from − 1.015 to − 0.420 ms/°C, indicating a consistent decrease in T2 values with increasing temperature. This suggests that the higher temperatures recorded at Site 2 are causing the observed reduction in T2 measurements.

DL CartiGram provided accurate in vivo measurements showing no significant differences from the T2 values measured with conventional CartiGram, while reducing scan time by 40%. Our primary analysis using paired *t* test, considered measurements within each region as independent, even though some regions originated from the same patients. To account for this potential confounder, we also performed a two-way ANOVA test, considering the regions of each cartilage compartment (femur and patella) as an additional factor in the analysis. The ANOVA results indicated that the variation in T2 measurements was significantly explained by differences in cartilage regions (*p* < 0.0001), while the reconstruction method had no significant effect on the measurements (*p* = 0.1), confirming that DL CartiGram is statistically equivalent to the conventional method. This further supports our hypothesis that DL reconstruction can effectively mitigate the noise enhancement typically associated with parallel imaging scan acceleration, resulting in precise and robust T2 maps. Recent work on accelerated T2 mapping techniques, such as GRAPPATINI, which employs parallel imaging combined with model-based reconstruction for the brain, knee, prostate, and liver imaging, reported lower accuracy in T2 measurements [30]. Specifically, for whole-brain imaging, GRAPPATINI demonstrated a relative error of 4.3% compared to reference Spin Echo measurements. This finding underscores the benefits of DL reconstruction in overcoming the limitations of accelerated T2 mapping methods.

The proposed semi-automatic pipeline for patient image processing constitutes a significant step towards the inclusion of quantitative T2 mapping as a cartilage health biomarker in the clinical setting, reducing the burden of manual postprocessing and improving the reproducibility of image analysis between operators. By adding registration and analysis steps, the proposed pipeline is also expected to be used for longitudinal follow-up studies or large cross-sectional studies, to help investigate cohorts at varying stages of OA and improve our overall understanding of this disease.

Conventional T2 mapping techniques require long scan times that could be impractical for cartilage imaging of the entire knee joint in a clinical setting. Accordingly, in this study, we had to focus sagittal acquisitions on one condyle of interest to save time. Having proven the accuracy of the fast DL T2 mapping acquisition, a whole sagittal acquisition covering both condyles can now be done within the same scan time as a single condyle using conventional CartiGram. Moreover, the scan time reduction in T2 mapping may help explore additional promising biomarkers.

Looking ahead, integrating relaxometry and morphometry (thickness/volume measurements) information into a single sequence presents a promising direction. A recent work illustrates the potential for combining these aspects to enhance diagnostic efficiency through a 5-min 3D double-echo steady-state (DESS) sequence [31]. Similarly, T2 shuffling enables sharp, multicontrast 3D FSE imaging with the ability to simultaneously generate morphological images and quantitative T2 maps from a single acquisition [32], offering an alternative pathway for efficient and comprehensive cartilage assessment. Furthermore, highly accelerated imaging techniques, such as those employing variable density k-space sampling [33] or simultaneous multi-slice (SMS) [34] acquisition, could further reduce scan times and improve imaging efficiency, which could significantly benefit both research and clinical practice for quantitative MRI. Extending DL-based acquisition and reconstruction techniques to advanced 3D relaxometry approaches could enable comprehensive quantitative cartilage assessment within clinically feasible scan times.

From an acquisition perspective, the QIBA profile recommends a 3D technique based on magnetization-prepared angle-modulated partitioned k-space spoiled gradient-echo snapshots (3D MAPSS), as it offers combined T1ρ and T2 capabilities. While these sequences are still undergoing development and validation, our study focused on the commercially available 2D MESE-based CartiGram technique, providing an accessible and clinically feasible platform for implementing DL-enhanced quantitative T2 mapping.

A critical factor in interpreting quantitative results is the fitting strategy used. Current MESE fitting methods, as employed in the Osteoarthritis Initiative (OAI), predominantly use mono-exponential models for T2 mapping [35–37], which are inherently sub-optimal as they do not account for stimulated echoes produced by RF slice-profile imperfections and B1 inhomogeneities. Although we addressed this by discarding the first echo to reduce errors from stimulated echoes, this approach sacrificed valuable data and reduced SNR efficiency. While these skipped-echo methods represent an improvement over standard exponential fitting, using models that account for all echo pathways would be more desirable. Recent advancements, such as T2 fitting using the extended phase graph (EPG) formalism [38], have shown promise in improving accuracy in MESE T2 mapping by more realistically simulating signal decay [39].

Our study had some limitations regarding scan-rescan experiments, as we did not test repeatability and reproducibility in healthy volunteers. Our investigation was limited to short-term reproducibility using phantoms, while understanding longitudinal minimum detectable variations would be critical for setting up multi-centre longitudinal studies using T2 mapping techniques. Additionally, we only included data from two sites, and expanding the study to include more platforms and different coil types, such as receive-only coils, could provide a more comprehensive assessment of T2 differences. With DL CartiGram now being rolled-out to more clinical studies, we expect such studies to be initiated very soon.

Regarding the patient cohort, 74% were male. However, the goal of this study was to assess the performance of DL CartiGram, and the cohort provided the necessary variability in cartilage health. Patients were primarily selected because it was feasible to perform both conventional and DL CartiGram sequences within the same session, addressing the time constraints of the study. The higher proportion of male patients is partly explained by the subgroup with autologous chondrocyte implantation, a procedure reported to have higher incidence in men at Site 1 [40]. In future longitudinal studies which focused on understanding cartilage degeneration or monitoring cartilage repair, it will be crucial to include balanced cohorts with healthy controls and a diverse demographic representation.

In conclusion, our findings demonstrate that, thanks to DL reconstruction, the accelerated T2 mapping technique can significantly shorten examination times, making T2 mapping feasible in the clinical settings. This approach provides consistent quantitative cartilage information with excellent repeatability and reproducibility across systems. Additionally, the proposed semi-automatic pipeline offers a streamlined workflow suitable for extensive clinical exploration, adoption, and potential future product implementation and commercialization.

## Data Availability

Data may be made available from the corresponding author upon reasonable request.
